# Supplementary material for: MetaRibo-Seq measures translation in microbiomes
Source: Nat Commun. 2020 Jun 29;11:3268. doi: 10.1038/s41467-020-17081-z (PMC7324362; doi:10.1038/s41467-020-17081-z)
Supplement: Supplementary file 10 — Supplementary Data 7 [file 41467_2020_17081_MOESM10_ESM.zip › File2/Confidence_VeryHigh_Taxonomy/78683_out.krona.html]

Javascript must be enabled to view this page.

members
magnitude
magnitudeUnassigned
count
unassigned
taxon
rank

78683\_out

5

2

SRS017103\_contig\_number\_contig-100\_23163.114581SRS142781\_contig\_number\_contig-100\_16056.16057

3
2
superkingdom

3
1239
phylum

3
186801
class

3
1
186802

SRS146888\_contig\_number\_contig-100\_4218.50063
order

family
186803
2

1
species

SRS063040\_contig\_number\_17037
1898203

genus
2039240
1

species
160404

SRS015264\_contig\_number\_11324
1
